# Papillary carcinoma thyroid with metastasis to ectopic cervical thymus

**DOI:** 10.1186/1477-7819-9-22

**Published:** 2011-02-18

**Authors:** Majid Mushtaque, Sameer H Naqash, Ajaz A Malik, Rayees A Malik, Samina A Khanday, Parwez S Khan

**Affiliations:** 1 Department of Surgery, Sheri Kashmir Institute of Medical Sciences, Soura, Srinagar, Jammu and Kashmir,190011, India; 2 Department of Pathology, Sheri Kashmir Institute of Medical Sciences, Soura, Srinagar, Jammu and Kashmir, 190011, India; 3 Department of Radiology, Sheri Kashmir Institute of Medical Sciences, Soura, Srinagar, Jammu and Kashmir, 190011, India

## Abstract

Papillary carcinoma of thyroid is the most common type of thyroid neoplasm which is usually confined to the thyroid and tends to metastasize to regional lymph nodes. Distant metastasis occur in up to 15% of cases. Thymic metastasis from any malignant carcinoma is extremely rare with only four cases reported in medical literature. We report a case of papillary carcinoma of thyroid metastasizing to ectopic cervical thymus which has not been previously reported.

## Introduction/Background

Papillary thyroid carcinoma is the most common neoplasm in the thyroid gland and accounts for about 70% of all thyroid carcinomas. This tumor peaks in the third or fourth decades of life, with female to male ratio ranging from 1.6:1 to 3:1 [1]. Thyroid cancers, especially papillary carcinoma, are more often found in patients with a history of external irradiation. Papillary carcinoma of thyroid may be sub clinical or may be present with asymptomatic thyroid mass or a nodule. Other symptoms like pain, difficulty breathing or swallowing, stridor, vocal cord paralysis, haemoptysis, rapid enlargement are rare. It commonly metastasizes to regional lymph nodes, but at the time of diagnosis, 10-15% of patients have distant metastases to the bones and lungs [2]. Other rare sites of distant metastasis are the brain, liver, and skin. [3]

The thymus is an important organ involved in cell-mediated immunological function, and to our knowledge, there has been only one case of papillary thyroid carcinoma with metastasis to thymus reported [4]. We report a case of papillary carcinoma of thyroid metastasizing to ectopic cervical thymus which has not been previously reported.

## Case Presentation

**Figure 1:**
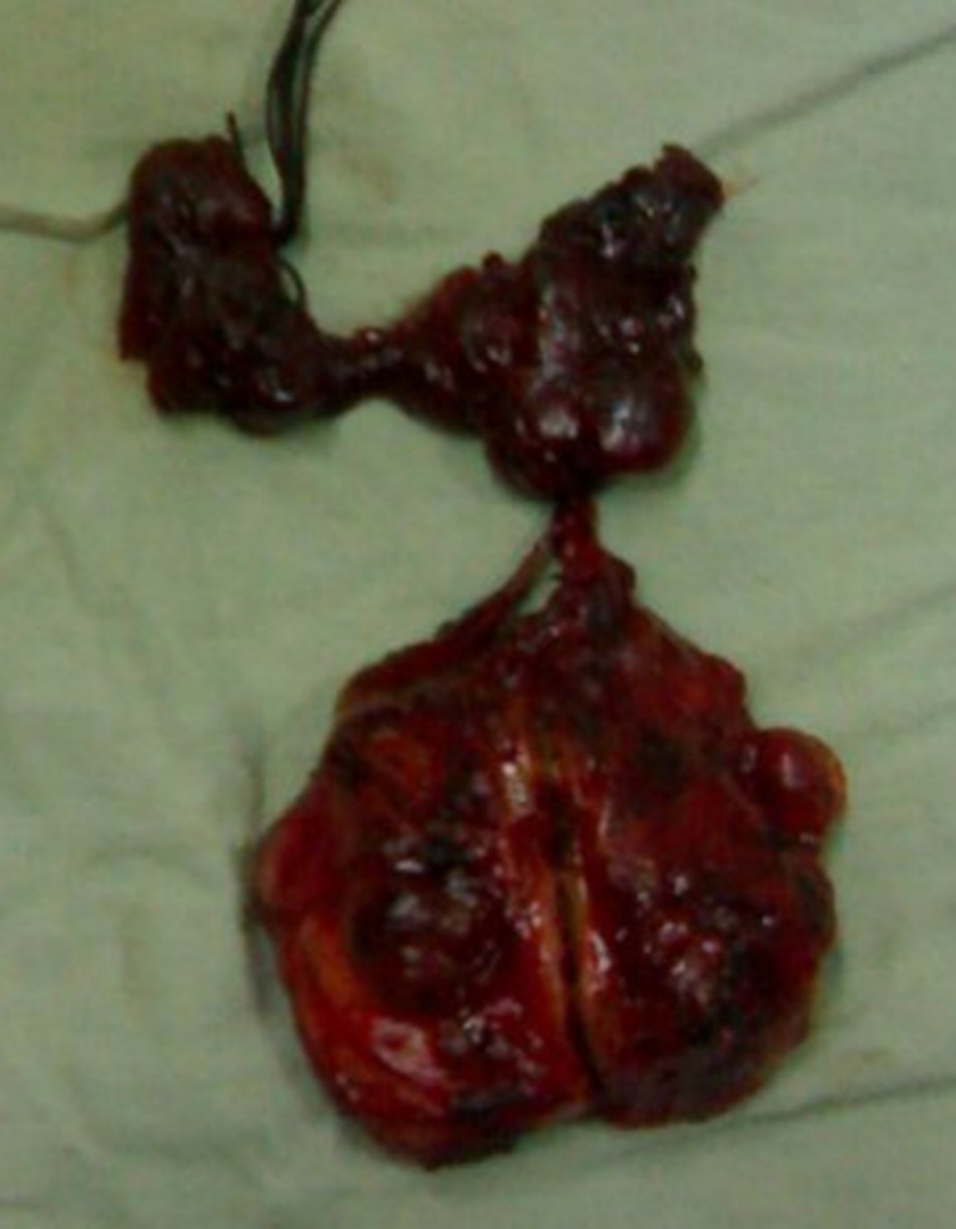
**Resected specimen of thyroid and thymus glands connected by a thyro-thymic ligament**.

**Figure 2:**
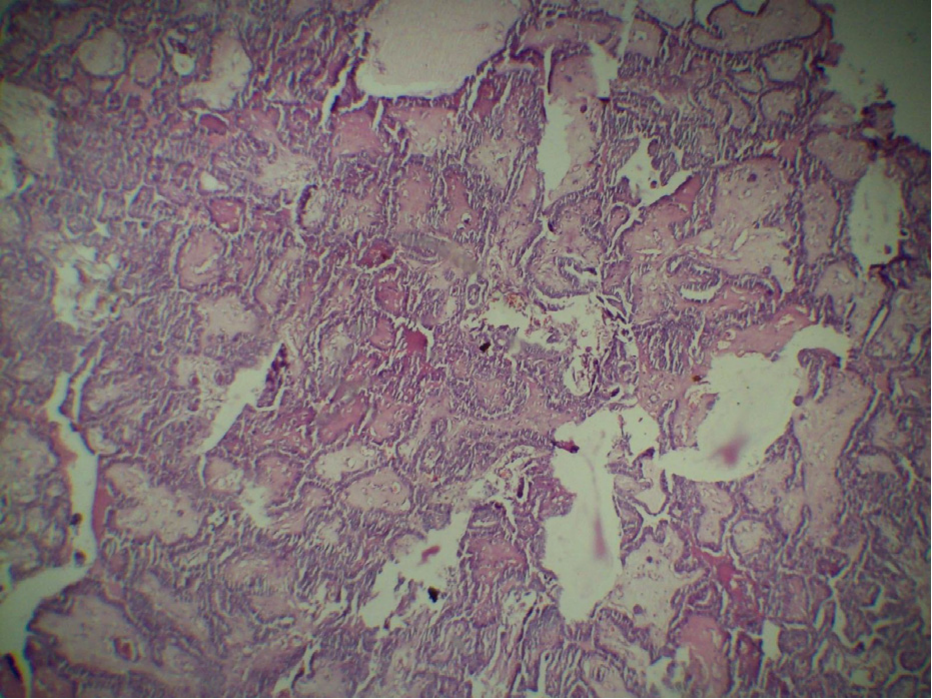
**Histopathological examination of thyroid showing papillary carcinoma (low power view)**.

**Figure 3:**
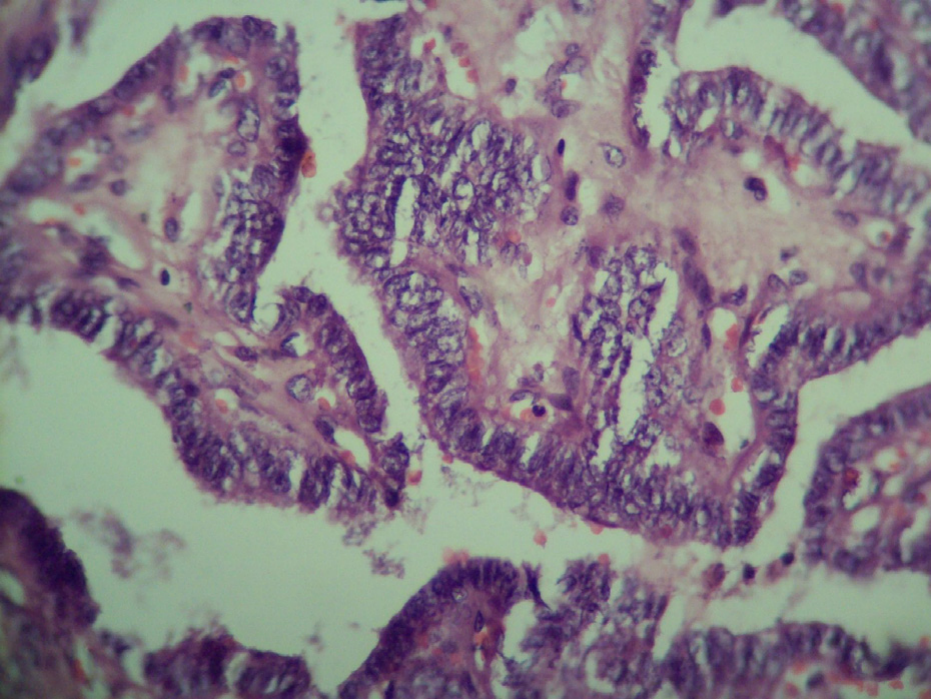
**Histopathological examination of thyroid showing papillary carcinoma (high power view)**.

**Figure 4:**
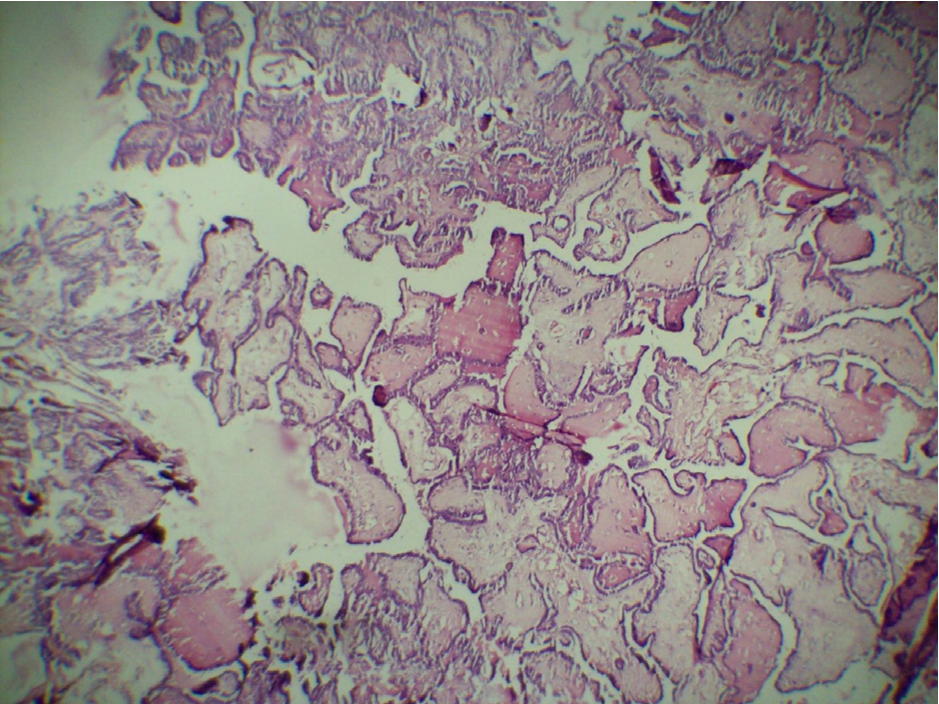
**Histopathological examination of thymus gland revealing metastasis from papillary carcinoma of thyroid (low power view)**.

**Figure 5:**
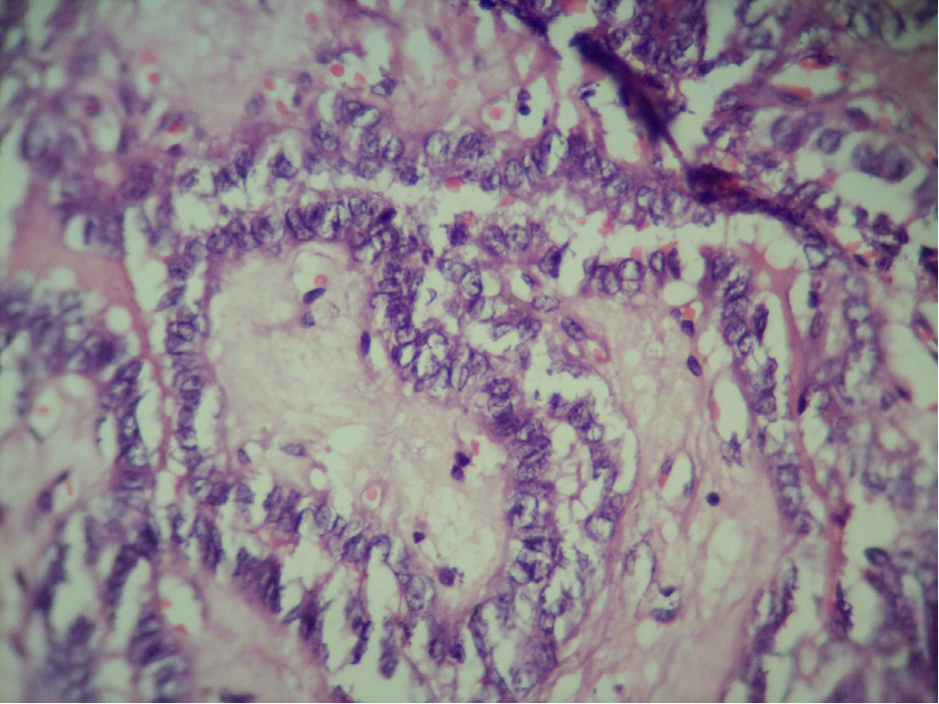
**Histopathological examination of thymus gland revealing metastasis from papillary carcinoma of thyroid (high power view)**.

A 42 year old female presented with a progressively enlarging painless swelling in the anterior part of the neck since 1 year. The only complaint was that of disfigurement. There was no other significant history. On examination, a single swelling was present in anterior neck, 13 × 8 cm in size, irregular in shape, extending vertically from thyroid cartilage above to supra sternal notch below and between two sternomastoid muscles. It was firm in consistency, moved freely with deglutition and had ill defined lower margin. There was no cervical lymphadenopathy. Examination of respiratory, cardiovascular, nervous systems and abdomen were normal. Thyroid function test was within normal range and FNAC (fine needle aspiration cytology) of the thyroid swelling revealed papillary carcinoma. Ultrasound of the neck documented a single mass in anterior neck, 12 × 8 cms in size with complex cystic and solid components without any associated cervical lymphadenopathy. The patient was planned for total thyroidectomy. Intra operative findings included slightly enlarged left lobe of thyroid (4 × 3 × 3 cm) with normal sized right lobe (3 × 3 × 2 cm). Another swelling (thymus) about 8 × 4 × 3 cm in size was found incidentally, adjacent to but separate from the thyroid at its lower margin and extending upto suprasternal notch. The thyroid and the thymus were only connected by a fibrous band (figure 1). Total thyroidectomy with thymectomy was performed. Post operative period was uneventful. The histopathological examination of the thyroid specimen revealed papillary carcinoma (figures 2 and 3) and the sections from attached mass (thymus) revealed multiple cysts with its tissue replaced by metastatic papillary carcinoma of thyroid (figures 4 and 5). Both tumors were reactive to thyroglobulin, keratin and CD3 confirming papillary carcinoma of thyroid with metastasis to ectopic cervical thymus. Radioiodine scan was done on follow up which did not detect any residual or any other metastatic disease. The patient is on regular follow up and is presently doing well.

Papillary carcinoma thyroid is the most common type of thyroid malignancy seen in the population especially females. This tumor usually has a good prognosis. It spreads via lymphatics and is commonly associated with enlarged cervical nodes. Bones and lungs are the usual sites of distant metastasis [2], however only one case of thymic metastasis has been reported till date [4].

As the thymus is an essential organ that controls the cellular immunity function, it has been considered almost impossible that a tumor could metastasize to the thymus. Although tumors almost never metastasize to the thymus, precise observation of the thymic structure has revealed that the thymus is not absolutely safe from tumor metastasis. The parenchyma of the thymus has a blood thymus barrier, which prevents the thymus from making direct contact with antigens or cancerous cells, thereby seemingly excluding the occurrence of cancer metastasis. However, the septum of the thymus is comprised of interlobular connective tissue with blood vessels, lymphoducts and nerves, which theoretically does not exclude the possibility of metastasis. Blood-thymus barrier is not as robust in the medulla of the organ as it is in the cortex. Also, it is not that blood-organ barrier can always prevent metastasis. Brain, eye and testis also have a blood-organ barrier and metastases in these organs have also been reported. Therefore, when the structure of the thymus is precisely analyzed, a remote possibility of the thymic metastasis from tumors is imaginable [5].

Embryologically, the thymus originates from the third pair of branchial pouches high in the neck during early foetal life and reaches its final destination in the mediastinum after a process of progressive decent. Rarely thymus fails to decent and appears as a remnant, implant, or accessory nodules any were along the cervical pathway, the most commonest site being at the level of thyroid gland [6]. Adult cases of ectopic thymus are exceedingly rare due to age related involution and replacement by fibro-adipose tissue. Ectopic thymus tissue like its normal counterpart may also undergo transformation to thymic hyperplasia or even thymic neoplasia [7].

There are very few reports of thymic metastasis including those from breast [5], prostate [8], testis [9] and thyroid cancers [4,10]. Our case represents a rarest case of papillary carcinoma thyroid with metastasis to the ectopic cervical thymus, which was found incidentally during thyroid surgery and was confirmed by histopathology and immunohistochemistry. The metastatic spread from thyroid cancer to ectopic thymus is presumed to be of haematogenous origin in absence of radiological or histological evidence of any local or nodal spread.

## Conclusion

Although it was earlier considered almost impossible that a tumor could metastasize to the thymus, a remote possibility of the thymic metastasis from tumors is imaginable and enlargement of ectopic cervical thymus should be considered in the differential diagnosis of anterior neck swellings.

## Consent

Written informed consent was obtained from the patient for publication of this case report and accompanying images.

## Competing interests

The authors declare that they have no competing interests.

## Authors' contributions

**MM **(Majid Mushtaque): Conception, Drafting, Revising the manuscript. Acquisition and Interpretation of data, Operating surgeon, Given final approval.

**SN **(Sameer H Naqash): Operating surgeon, Interpretation and Acquisition of data and Given final approval.

**AM**(Ajaz A Malik): Operating surgeon, Revising the manuscript, Interpretation of data and Given final approval.

**RM**(Rayees A Malik): Pathological examination of the specimen and Given final approval.

**SK**(Samina A Khanday): Did Sonographic examination of the patient, Interpretation and Acquisition of data and Given final approval.

**PK**(Parwez S Khan): Interpretation and Acquisition of data, Drafting and Given final approval.
